# Crooks Fluctuation Theorem for Single Polymer Dynamics in Time-Dependent Flows: Understanding Viscoelastic Hysteresis

**DOI:** 10.3390/e24010027

**Published:** 2021-12-24

**Authors:** Yuecheng Zhou, Folarin Latinwo, Charles M. Schroeder

**Affiliations:** 1 Department of Materials Science and Engineering, University of Illinois at Urbana-Champaign, Urbana, IL 61801, USA; ypz@stanford.edu; 2 Beckman Institute for Advanced Science and Technology, University of Illinois at Urbana-Champaign, Urbana, IL 61801, USA; 3 Department of Chemical and Biomolecular Engineering, University of Illinois at Urbana-Champaign, Urbana, IL 61801, USA; Folarin.Latinwo@synopsys.com

**Keywords:** fluctuation theorems, nonequilibrium thermodynamics, polymer dynamics, conformation hysteresis, viscoelasticity

## Abstract

Nonequilibrium work relations have fundamentally advanced our understanding of molecular processes. In recent years, fluctuation theorems have been extensively applied to understand transitions between equilibrium steady-states, commonly described by simple control parameters such as molecular extension of a protein or polymer chain stretched by an external force in a quiescent fluid. Despite recent progress, far less is understood regarding the application of fluctuation theorems to processes involving *nonequilibrium* steady-states such as those described by polymer stretching dynamics in nonequilibrium fluid flows. In this work, we apply the Crooks fluctuation theorem to understand the nonequilibrium thermodynamics of dilute polymer solutions in flow. We directly determine the nonequilibrium free energy for single polymer molecules in flow using a combination of single molecule experiments and Brownian dynamics simulations. We further develop a time-dependent extensional flow protocol that allows for probing viscoelastic hysteresis over a wide range of flow strengths. Using this framework, we define quantities that uniquely characterize the coil-stretch transition for polymer chains in flow. Overall, generalized fluctuation theorems provide a powerful framework to understand polymer dynamics under far-from-equilibrium conditions.

## 1. Introduction

Understanding the dynamics of soft materials and complex fluids is of fundamental interest to materials scientists, engineers, and rheologists [1]. Soft materials processing often involves highly nonequilibrium conditions that are difficult to model using the standard framework of equilibrium thermodynamics [2]. For example, processes such as flow-guided printing of semiconducting polymers [3], flow-assisted nonequilibrium assembly of hybrid synthetic oligopeptides [4,5], or flow-induced phase separation of colloidal particles [6] are governed by an interplay between far-from-equilibrium structure and dynamics. However, bulk material properties and the macroscopic viscoelastic response are often determined by the molecular properties of soft materials. In order to understand and effectively control material properties during flow processing, it is essential to develop new molecular-level approaches that connect the nonequilibrium energetics of soft materials to transient flow conditions. From this view, the development of new molecular-level thermodynamic frameworks for flowing systems will allow researchers to fundamentally understand the nonequilibrium processes governed by thermodynamics and rheology [2].

Single molecule techniques allow for a direct observation of individual polymer chains in flow, which provides access to the entire molecular ensemble under nonequilibrium conditions [7]. In this way, single molecule experiments allow for the characterization of different micro-states or molecular sub-populations in flow, which allows fluctuation theorems to be applied to understand nonequilibrium energetics and processes. Fluctuation theorems refer to a class of thermodynamic identities that describe the probability distribution of quantities such as work, heat, or entropy changes along stochastic trajectories [8]. In the context of fluctuation theorems or work relations, transitions between different thermodynamic states of a system can be analyzed. Hatano and Sasa derived an extended form of the second law to analyze transitions between thermodynamic steady states [9]. Jarzynski derived an equality that allows equilibrium-free energy differences to be determined from nonequilibrium work measurements [10]. Recently, an expression was developed to determine the nonequilibrium thermodynamic quantities for systems near equilibrium [11]. From this view, single molecule techniques provide an ideal platform to understand stochastic trajectories and to determine fundamental equilibrium and nonequilibrium thermodynamic quantities. By applying nonequilibrium work relations to the trajectories of single polymers in an imposed fluid flow, Schroeder and coworkers demonstrated the ability to determine polymer chain elasticity from the nonequilibrium stretching trajectories of polymer molecules [12] and further determined the equilibrium-free energy landscape for polymer chains in strong flows [13]. Moreover, Schroeder and coworkers also determined nonequilibrium thermodynamic quantities for polymers in flow and demonstrated the ability to determine polymer relaxation times purely from nonequilibrium stretching dynamics in flow [14].

The Crooks fluctuation theorem (CFT) allows the free energy difference between two states (*A* and *B*) to be determined solely from work distributions obtained from repeated forward processes (A→B) and backward processes (B→A) provided that the transitions are initialized from steady-state and are reversible [15]. Formally, the CFT is given as:

(1) $$\begin{array}{c} \frac{P_{F}\left(W\right)}{P_{B}\left(-W\right)}=\exp\left(\frac{W-\Delta F}{k_{B}T}\right) \end{array}$$

where $P_{F}\left(W\right)$ and $P_{B}\left(-W\right)$ are the probability distributions of the work performed in the forward (*W*) and backward process (−W), respectively, $k_{B}T$ is the Boltzmann temperature, and ΔF is the Helmholtz free energy change between states *A* and *B* [15]. Using the CFT, the free energy change for a process ΔF is determined where $P_{F}\left(W\right)=P_{B}\left(-W\right)$ or exactly where the forward and backward work distributions intersect. In practice, the CFT has been applied to understand the thermodynamics of micron-sized colloidal particles immersed in water at millisecond timescales [16]. Moreover, the CFT was also used to study the energetics of biological molecules and biophysical systems. For example, the *equilibrium*-free energy differences between the unfolded and folded states of an RNA hairpin were determined by directly measuring work distributions from force-extension measurements using an optical trap [17]. However, there has been far less progress in using the CFT to determine the free energy differences between *nonequilibrium* steady-states involving the flow processing of polymeric materials. Prior work applied the CFT to an elastic dumbbell model of polymers in an extensional flow [18] or a two-dimensional inviscid and incompressible flow on a rectangular domain [19], though these demonstrations only considered simulations of model systems. Here, we use a combination of experiments and simulations to show that the CFT can be used to determine nonequilibrium-free energy differences for polymeric materials in flow, specifically focusing on a quantitative understanding of viscoelastic hysteresis. We note for the nonequilibrium steady-states described in this article, an effective Helmholtz free energy $F^{*}$ rather than Helmholtz-free energy *F* is used (*vide infra*) [14].

Soft polymeric materials generally exhibit history-dependent deformation behavior in flow [1,20,21]. For polymer melts in shear flow, when the shear rate is gradually increased from an initial value of ${\dot{\gamma }}_{min}$ to a value of ${\dot{\gamma }}_{max}$ and then gradually returned to the initial value of ${\dot{\gamma }}_{min}$, the shear stress $\tau_{xy}$ measured during this process exhibits ‘hysteresis loops’. In particular, when the system transitions from ${\dot{\gamma }}_{min}$ to ${\dot{\gamma }}_{max}$, the ‘upward curve’ of $\tau_{xy}$ is distinctly different than the ‘downward curve’ when the system travels back from ${\dot{\gamma }}_{max}$ to ${\dot{\gamma }}_{min}$. These hysteresis loops are known as viscoelastic hysteresis or stress-strain hysteresis in polymer melts and are found to arise due to the polymer normal stress differences in flow described by the Bird–Carreau model [22,23]. In dilute solutions, a stress-birefringence viscoelastic hysteresis was also observed in stretch-relaxation cycles of single polymers in uniaxial extensional flow [24,25]. The stress-birefringence viscoelastic hysteresis was found to arise due to a broad non-Gaussian distribution of molecular extension during the stretch phase, where the highly stretched chains dominantly contribute to the stress, but the birefringence signal is dominated by more weakly stretched chains. Therefore, the stress is generally larger during the transient startup phase of extensional flow than the relaxation phase [24]. Recently, an analogous rheological hysteresis was also reported in soft glassy materials [26].

A different type of hysteresis behavior known as polymer conformation hysteresis occurs for high molecular weight (MW) polymer chains in extensional flow [27]. Early work by de Gennes predicted the existence of a coil-stretch hysteresis for polymer chains in a narrow range of flow strength in extensional flow [28]. Unlike stress-strain hysteresis for polymer melts in flow, polymer conformation hysteresis was predicted to arise due to intramolecular hydrodynamic interactions (HI) for polymer chains in flow, which give rise to a conformation dependent drag force in flow. In this way, the fluid exerts a larger frictional grip on fully stretched chains compared to polymer coils, resulting in conformational hysteresis near the coil-stretch transition in strong flows such as extensional flows [28]. Polymer conformation hysteresis was experimentally observed for extremely large DNA molecules in planar extensional flow using single molecule fluorescence microscopy [27]. Single molecule experiments were complemented by Brownian dynamics (BD) simulations of large polymer chains that confirmed the existence of coil-stretch hysteresis for high molecular polymers in flow [27,29]. Numerical simulations further demonstrated that a large difference in conformation-dependent drag for polymer molecules in a coiled versus stretched state is necessary for the observation of this form of hysteresis [29]. Hence, high molecular weight polymer molecules with significant intramolecular HI exhibit a clear history-dependent conformation hysteresis in extensional flow. In semidilute polymer solutions, polymer conformation hysteresis in extensional flow was found to depend on concentration based on results using a combination of bulk extensional rheometry experiments and BD simulations [30,31]. In semidilute polymer solutions, the hysteresis window significantly widens with increasing concentration, reaching a maximum at the critical overlap concentration $c^{*}$, and decreases before vanishing at a high polymer concentration in the semidilute regime. Recent atomistic simulations of polyethylene melts have shown that entangled polymer chains also exhibit a coil-stretch hysteresis at intermediate flow strength [32,33]. Interestingly, hysteresis in this system is reflected by a bimodal distribution of highly stretched and coiled chains, with occasional transitions between the two states. The duality in chain conformation leads to a flow-induced phase separation into ellipsoidal domains of coiled molecules surrounded by thin sheets of highly stretched molecules.

In addition to the dramatic conformation hysteresis exhibited by high MW polymers in extensional flow, intramolecular HI also plays a key role in the dynamics of lower MW polymer chains in strong flows, quantitatively altering their flow dynamics and resulting in a critical ‘slowing-down’ of transient stretching and relaxation dynamics in the vicinity of the coil-stretch transition due to a large number of available states and large chain fluctuations [34]. Although conformational hysteresis is generally not observed for low MW polymer chains, intramolecular HI significantly alters chain dynamics even for low MW polymers.

Direct observation of polymer conformation hysteresis in dilute solutions requires large accumulated fluid strains to be imposed on single polymer chains under controlled steady flow conditions [27]. Prior single molecule experiments used a slow transition rate when stepping between different flow strengths to ensure that single polymers fully ‘relax’ to the flow deformation rate in experiments [27]. The observation window for polymer conformation hysteresis is limited to a narrow range of flow strengths for ultra-dilute solutions [35] and requires reasonably long observation times. The effective nonequilibrium polymer conformational energy exhibits a double-welled potential near the coil-stretch transition with an effective energy barrier larger than thermal energy, resulting in high MW polymers becoming trapped in effective energy minima and exhibiting conformation hysteresis over finite observation times [36]. Kramers hopping theory was used to describe the hopping rate of polymer chains between different conformational energy states, which shows that the hopping rate is inversely proportional to the polymer chain length [37]. In this way, polymer conformation hysteresis can be unambiguously defined as occurring when the inverse hopping rate is much larger than the experimental observation times [37]; the inverse hopping rates are typically astronomically large for high MW polymer chains. Of course, if an infinite observation time were possible, polymer chains would evolve to the lowest conformational energy state [36]. Nevertheless, in practical situations involving flow processing, high MW chains become kinetically trapped for extremely long times in stable stretched or coiled conformations near the coil-stretch transition, which is a signature of polymer conformational hysteresis.

In this article, we apply the Crooks fluctuation theorem (CFT) to understand polymer dynamics in time-dependent flows, specifically focusing on the history-dependent dynamic behavior of single polymers in strong flows. In particular, we focus on understanding viscoelastic hysteresis in low MW polymers at the molecular-level (i.e., we only consider viscoelastic hysteresis and not conformational hysteresis, as discussed below). We present a new time-dependent extensional flow protocol that allows for characterization of rate-dependent viscoelastic hysteresis observable over a broad range of flow conditions. Using this approach, we define quantities that uniquely characterize the coil-stretch transition for polymer chains in strong time-dependent flows. We also apply the CFT to understand the nonequilibrium stretching dynamics of single polymers in time-dependent flows. We directly determine free energy differences between nonequilibrium steady-states (NESSs) by calculating work distributions from far-from-equilibrium transient properties. In general, reasonable agreement between single molecule experiments and BD simulations is observed using this framework. Overall, our work demonstrates a new route for quantitatively understanding the nonequilibrium thermodynamic properties of soft materials under nonequilibrium flow conditions.

## 2. Methods

### 2.1. Flow Protocol for Using Fluctuation Theorems

In order to study the time-dependent viscoelastic behavior of polymers at the molecular level [20], a time-dependent flow forcing function is required. Recently, large amplitude oscillatory extensional (LAOE) flow was used to understand the dynamics of polymers at the single molecule level [38,39]. LAOE is a highly transient and time-dependent flow field that allows for determination of molecular stretch-strain rate curves known as single molecule Lissajous curves. Interestingly, Lissajous curve shapes were interpreted in the context of polymer chain conformation over a wide range of dimensionless flow strength (Weissenberg number, *Wi*) and dimensionless flow frequency (Deborah number, *De*) [38]. In this article, we present a different time-dependent flow protocol for studying single polymer dynamics in extensional flow. Using this protocol, we characterize polymer dynamics and viscoelastic hysteresis over a wide range of flow strength, Wi, and flow transition rate, De, using a combination of single molecule experiments and BD simulations.

**Figure 1:**
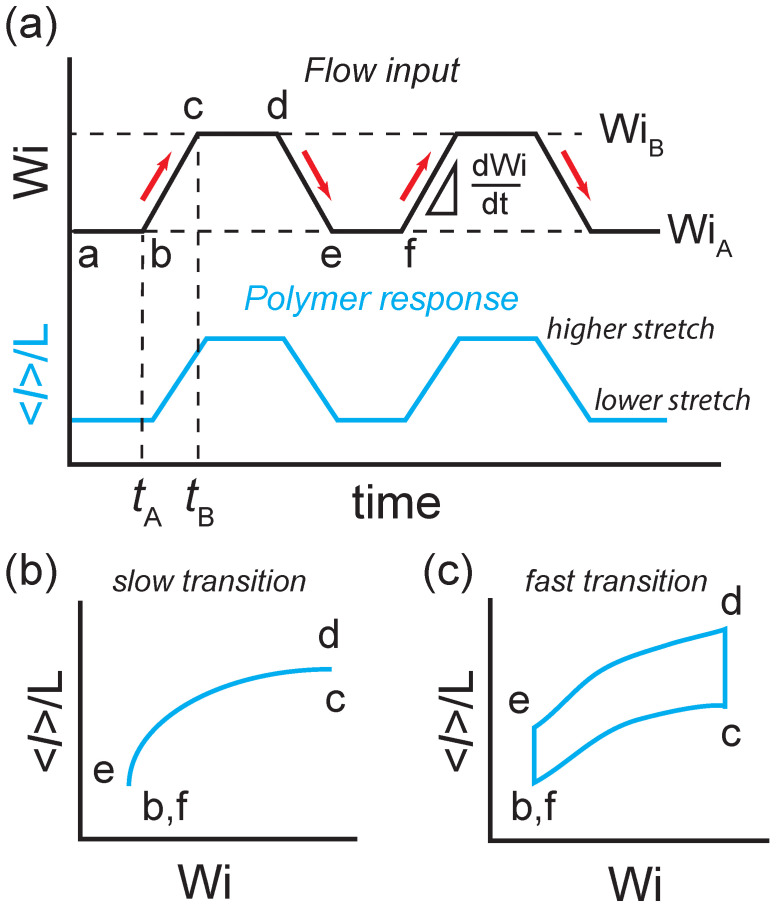
Time-dependent flow protocol for single polymers in planar extensional flow used in single polymer experiments and Brownian dynamics simulations. (**a**) Time trajectory of the extensional flow input (top curve) and ensemble-averaged polymer fractional extension 〈l〉/L resulting from the flow forcing function (bottom curve). Rate-dependent stretch-strain rate loops for ensemble-averaged polymer fractional extension 〈l〉/L versus flow strength Wi in response to (**b**) a slow transition rate from $Wi_{A}$ to $Wi_{B}$, and (**c**) a fast transition rate from $Wi_{A}$ to $Wi_{B}$.

The time-dependent extensional flow protocol used for single polymers is shown in Figure 1. Here, polymer chains are first allowed to relax to a nonequillibrium steady-state at an imposed initial flow strength $Wi_{A}$ in extensional flow (Figure 1a, a→b). Next, the polymer is transitioned to a second flow strength, $Wi_{B}$ at a finite transition rate dWi/dt (Figure 1a, b→c). Following this forward transition, the polymer is then allowed to relax to a second nonequilibrium steady-state at the new flow strength $Wi_{B}$ (Figure 1a, c→d). The polymer is then transitioned again from the nonequilibrium steady-state at $Wi_{B}$ back to $Wi_{A}$ at a finite transition rate −dWi/dt (Figure 1a, d→e). Following the backward transition, the polymer is once again allowed to reach nonequilibrium steady-state at $Wi_{A}$ (Figure 1a, e→f).

This process is performed repeatedly such that an ensemble of molecular deformation trajectories in response to the flow protocol is observed. Based on the molecular response to the forward ($Wi_{A}\to Wi_{B}$) and backward ($Wi_{B}\to Wi_{A}$) transitions, the ensemble-averaged polymer fractional extension 〈l〉/L with respect to flow strength Wi (Figure 1b,c) and the corresponding nonequilibrium work distributions are determined. Using this flow protocol, the ensemble-averaged extension allows for characterization of viscoelastic hysteresis, and the work distributions allow for the determination of the nonequilibrium-free energies of single polymers as a function of flow strength.

### 2.2. Brownian Dynamics Simulations

We use a free-draining coarse-grained bead-spring polymer chain model and Brownian dynamics (BD) simulations to model the dynamics of single DNA molecules in time-dependent planar extensional flow (Figure 1a). In these simulations, a single polymer chain is modeled as $N_{b}$ beads connected by $N=N_{b}-1$ springs. A force balance yields an inertialess Langevin equation of motion [40]:

(2) $$\begin{array}{c} dR=\left[U+\frac{1}{k_{B}T}D\cdot F^{s}+\frac{\partial }{\partial R}\cdot D\right]dt+\sqrt{2}B\cdot dW \end{array}$$

where $R\left(r_{k}\right)$ is the vector of bead position vectors $r_{k}$, U is the velocity field, D is the diffusion tensor, and $F^{s}$ is the entropic elasticity from the springs. To account for thermal motion of the chain in a continuum solvent, B is chosen to satisfy the fluctuation-dissipation theorem, such that $D=B\cdot B^{T}$, and dW is determined from a Gaussian distribution with zero mean and variance dt. For our time-dependent planar extensional flow protocol, the velocity field U=κ(t)·R with $\kappa \left(t\right)=\kappa_{ij}\left(t\right)=\dot{\epsilon }\left(t\right)\left(\delta_{i1}\delta_{j1}-\delta_{i2}\delta_{j2}\right)$, where $\dot{\epsilon }\left(t\right)$ is the applied time-dependent strain rate and $\delta_{ij}$ is the Kronecker delta.

Based on the time-dependent strain rate, the Weissenberg number is defined as $Wi=\dot{\epsilon }\tau_{R}$, where $\tau_{R}$ is the longest polymer relaxation time [12]. Here, we use a single-mode dumbbell model with two beads ($N_{b}=2$) connected by an entropic spring with worm-like chain (WLC) elasticity [41] to model the behavior of λ-DNA molecules in response to the time-dependent extensional flow protocol. Prior work has shown that single-mode bead-spring chains (dumbbells) accurately capture the qualitative dynamics of linear polymer chains in extensional flow [36]. In this way, we are neglecting intramolecular HI in our simulations, and the dumbbell model is considered to be free-draining. As mentioned above, the present work is focused on understanding viscoelastic hysteresis, which arises due to viscoelastic effects of single polymer chains in response to a time-dependent strain rate deformation, rather than conformational hysteresis. Of course, intramolecular HI is known to exaggerate viscoelastic hysteresis effects, and in the limit of high MW polymers, will induce conformational hysteresis in extensional flow. Nevertheless, a free-draining dumbbell model is sufficient to capture the underlying physics of viscoelastic hysteresis for low MW polymer chains such as λ-DNA molecules in extensional flow [36]. The contour length *L* of fluorescently labeled λ-DNA molecules is taken to be 21.5 μm in simulations and experiments [7]. Additional simulation details are described in prior work [14].

With regards to fluctuation theorems and work relations, our system is defined as a single polymer molecule in a time-dependent planar extensional flow, and the control parameter that defines the state of the system is the dimensionless flow strength, Wi. In this way, single polymer chains are transitioned between nonequilibrium steady states (NESSs) in extensional flow because the control parameter is the flow rate instead of the polymer extension, as discussed in prior work [14]. The transition time between State A ($Wi_{A}$) and State B ($Wi_{B}$) is nondimensionalized to obtain a Deborah number $De=\tau_{R}/\left(t_{B}-t_{A}\right)$. In this way, the Deborah number De describes the transition rate between NESSs with different flow strengths, defined relative to the polymer relaxation time, which is an intrinsic property of the polymer chain. Therefore, a small De indicates a slow transition rate between NESSs such that polymers have sufficient time to respond to the flow deformation, whereas a large De indicates a fast transition rate between NESSs such that polymers may not have sufficient time to respond to the flow deformation.

### 2.3. Single Molecule Experiments

Double-stranded λ-DNA (48.5 kbp, New England Biolabs, Ipswich, MA, USA) is used for single molecule imaging. λ-DNA molecules are fluorescently labeled with an intercalating dye YOYO-1 (Invitrogen, Thermo Fisher, Waltham, MA, USA) at a dye-to-base pair ratio of 1:4 for >1 h in a dark at room temperature. Fluorescently labeled λ-DNA is added to an imaging buffer containing 30 mM Tris/Tris-HCl (pH 8.0), 2 mM EDTA, 5 mM NaCl, glucose (5 mg/mL), glucose oxidase (0.05 mg/mL), catalase (0.01 mg/mL), and 4% *v*/*v* β-mercaptoethanol. The imaging buffer solvent viscosity is increased to 48.5 ± 0.1 cP at 23 °C by addition of sucrose (60% *w*/*w*). Glucose oxidase/catalase is used as a coupled enzymatic oxygen scavenging system to suppress photobleaching and photocleaving of the fluorescently labeled DNA. The concentration of DNA is ultra-dilute (10${}^{-5}$ $c^{*}$), and experiments are performed in the absence of polymer-polymer interactions.

Imaging is performed using an inverted epifluorescence microscope (IX71, Olympus, Tokyo, Japan) illuminated with a 100 W mercury arc lamp (USH102D, UShio, Tokyo, Japan) directed through a 3% neutral density filter (Olympus), a 482 ± 18 nm band-pass excitation filter (FF01-482/18–25, Semrock, New York, NY, USA), and a 488 nm single-edge dichroic mirror (Di01-R488-25 × 36, Semrock). Fluorescence emission is collected by a 1.45 NA, 100× oil immersion objective lens (UPlanSApo, Olympus), and a 488 nm long pass filter (BLP01-488R-25, Semrock) is used in the detection path. Finally, images are acquired by an Andor iXon electron-multiplying charge coupled device camera (512 × 512 pixels, 16 μm pixel size) under frame-transfer mode at a frame rate of 33 Hz.

The Stokes trap is used to precisely position and manipulate polymer molecules in flow [38,39,42,43,44,45]. The Stokes trap uses model predictive control (MPC) to confine fluorescently labeled λ-DNA molecules near the stagnation point of a planar extensional flow for long observation times, as previously described [42]. In brief, a microfluidic cross-slot device is used to generate a planar extensional flow, and the Stokes trap is used to confine single polymer chains in precisely defined time-dependent extensional flows (Figure 1a). The imposed strain rate $\dot{\epsilon }$ is determined experimentally using particle tracking velocimetry (PTV). Additional details regarding implementation of the Stokes trap to generate complex time-dependent flows for single polymer dynamics are described in prior work [38,39].

## 3. Results and Discussion

### 3.1. Viscoelastic Hysteresis

We began by studying dynamic polymer stretching behavior using the time-dependent extensional flow protocol shown in Figure 1. Here, the Stokes trap is experimentally implemented using a four-channel cross-slot microfluidic device with pressurized inlets connected to four computer-controlled pressure regulators. First, a single DNA molecule is confined in extensional flow at State A (at $Wi_{A}$) for residence times at least $5\tau_{R}$–$10\tau_{R}$ to ensure that the polymer has reached a NESS (Figure 1a, a→b). The flow strength is then gradually increased from $Wi_{A}$ to $Wi_{B}$ by increasing the pressure at the inlets with a constant programmed rate (Figure 1a, b→c). The polymer chain is then maintained at State B (at $Wi_{B}$) for at least $5\tau_{R}$–$10\tau_{R}$ (Figure 1a, c→d) before the flow rate is transitioned back from $Wi_{B}$ to $Wi_{A}$ at the same transition rate (Figure 1a, d→e). Finally, the polymer chain is again held at $Wi_{A}$ to reach a different NESS (Figure 1a, e→f). During this process, single DNA molecules are trapped near the stagnation point of the extensional flow in the microfluidic device using a feedback controller that applies small pressures to the pressurized inlets. The feedback control pressures are negligible compared to the primary pressure used to generate the time-dependent extensional flow [38,39].

In this time-dependent flow protocol, when the transition rate between $Wi_{A}$ and $Wi_{B}$ is slow (e.g., small De), the polymer has sufficient time to relax to the flow deformation, and the polymer extension generally follows the same forward and backward transition from $Wi_{A}\to Wi_{B}$ and $Wi_{B}\to Wi_{A}$, respectively. Under these conditions, we expect no viscoelastic hysteresis loop to be observed (Figure 1b). However, when the transition rate between $Wi_{A}$ and $Wi_{B}$ is fast (e.g., large De), the polymer chain generally does not have sufficient time to relax to the flow deformation. Hence, after the polymer transitions from $Wi_{A}\to Wi_{B}$, it continues to equilibrate to the NESS at a higher flow strength at $Wi_{B}$, and the polymer extension continues to increase (Figure 1c, c→d). We define this hysteresis in polymer extension at a higher flow strength as the ‘stretch-lag’. Similarly, after the polymer transitions back from $Wi_{B}\to Wi_{A}$, it continues to equilibrate to the NESS at lower flow strength at $Wi_{A}$, and the polymer extension continues to decrease (Figure 1c, e→f). We define this hysteresis in polymer extension at a lower flow strength as the ‘coil-lag’. In this way, a hysteresis loop emerges due to the ‘stretch-lag’ and ‘coil-lag’, which constitutes the right-hand side and left-hand side of the hysteresis loop, respectively (Figure 1c).

**Figure 2:**
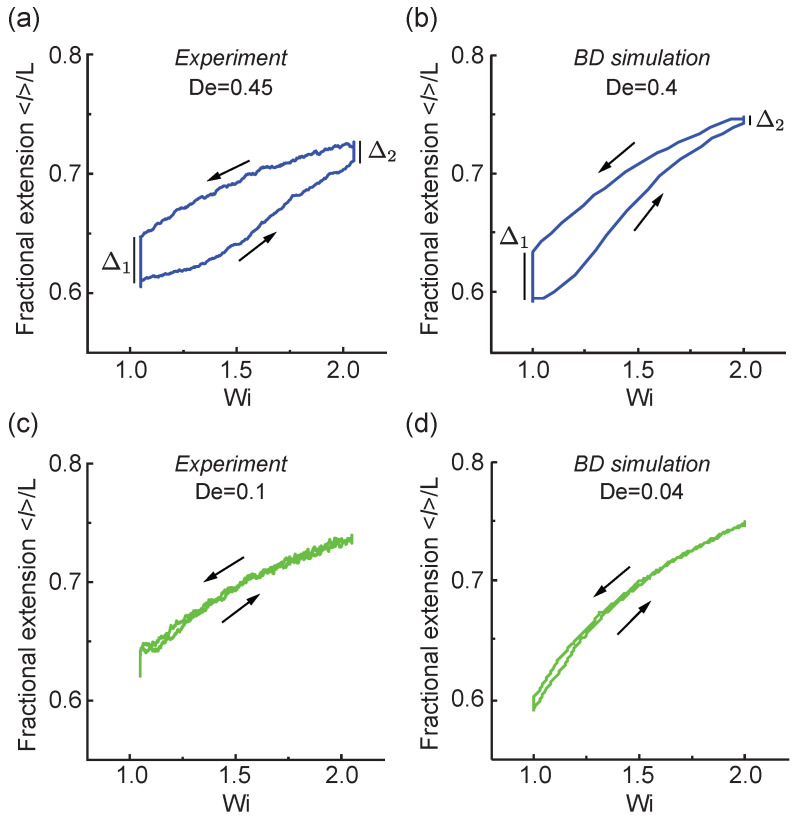
Rate-dependent polymer stretch loops transitioning from $Wi_{A}=1$ to $Wi_{B}=2$ with a relatively large transition rate De≈0.4 from (**a**) single molecule experiments and (**b**) Brownian dynamics (BD) simulations, and intermediate transition rates De≈0.1 from (**c**) single molecule experiments and (**d**) BD simulations. Here, $\Delta_{1}$ indicates the ‘coil-lag’ and $\Delta_{2}$ indicates the ‘stretch-lag’. Arrows indicate the direction of the forward transition ($Wi_{A}\to Wi_{B}$) and backward transition ($Wi_{B}\to Wi_{A}$). The experimental ensembles contain n=50 and n=38 molecular traces for De=0.45 and De=0.1, respectively. All simulation ensembles contain n=500 molecular traces.

Our results show a strong rate-dependent behavior in the ensemble-averaged polymer fractional extension 〈l〉/L when transitioning between flow rates Wi above the polymer coil-stretch transition (CST). Figure 2a,b show results from experiments and BD simulations for polymer stretching between $Wi_{A}=1$ and $Wi_{B}=2$, which corresponds to initial and final flow rates above the CST. As shown in Figure 2a,b, under a relatively fast transition rate of De≈0.4, polymers are stretched from 〈l〉/L≈0.6 to 〈l〉/L≈0.7 following the trajectory along the right arrow. Upon reaching the final $Wi_{B}$, the polymer chain is maintained at a constant flow strength to fully equilibrate to the nonequilibrium state at $Wi_{B}$. At the higher flow strength of Wi=2 where 〈l〉/L≈0.7, the polymer fractional extension continues to increase with a hysteretic ‘stretch-lag’ value of $\Delta_{2}$.

After equilibrating at the nonequilibrium steady state at Wi=2, the flow protocol is continued, and the flow rate decreases at a given rate (De≈0.4) to the initial flow rate at Wi=1 (Figure 2a,b). During this step, the polymer extension decreases from 〈l〉/L≈0.7 to 〈l〉/L≈0.65 following the decreasing flow strength as indicated by the trajectory shown by the left arrow. At a lower flow strength of Wi=1, the polymer extension continues to drop from 〈l〉/L≈0.65 back to the starting point at 〈l〉/L≈0.6 with a hysteretic value of $\Delta_{1}$, which corresponds to the ‘coil-lag’ value following the definition above. The coil (stretch)-lag refers to the absolute maximum difference between the forward and reverse average fractional extensions at the lower (higher) flow strength. To this end, this process forms the viscoelastic hysteresis loop at a fast transition time because the polymer chain does not have enough time to respond to the flow deformation, resulting in a lag behind the time-dependent extensional flow input.

Under slow or intermediate transition rates of De≈0.1 (Figure 2c,d), the polymer extension increases and decreases between 〈l〉/L≈0.6 and 〈l〉/L≈0.7 following the flow input, as indicated by the directions of the arrows. At these slower transition rates, the polymer chain has sufficient time to relax and respond and to the transient flow deformation such that the ‘stretch-lag’ and ‘coil-lag’ are negligible. In this way, the viscoelastic hysteresis loop collapses to a single curved line, and the transient polymer extension closely follows the flow deformation. Results from single molecule experiments show reasonable quantitative agreement with BD simulations. In general, BD simulations tend to predict a slightly larger polymer extension at high flow strength and a slightly lower polymer fractional extension at low flow strength, which could arise due to the absence of intramolecular HI and excluded volume interactions in the free-draining model. Nevertheless, the free-draining dumbbell model with WLC elasticity is observed to qualitatively capture the dynamics of single DNA polymers (e.g., λ-DNA molecules) under the time-dependent flow protocol.

**Figure 3:**
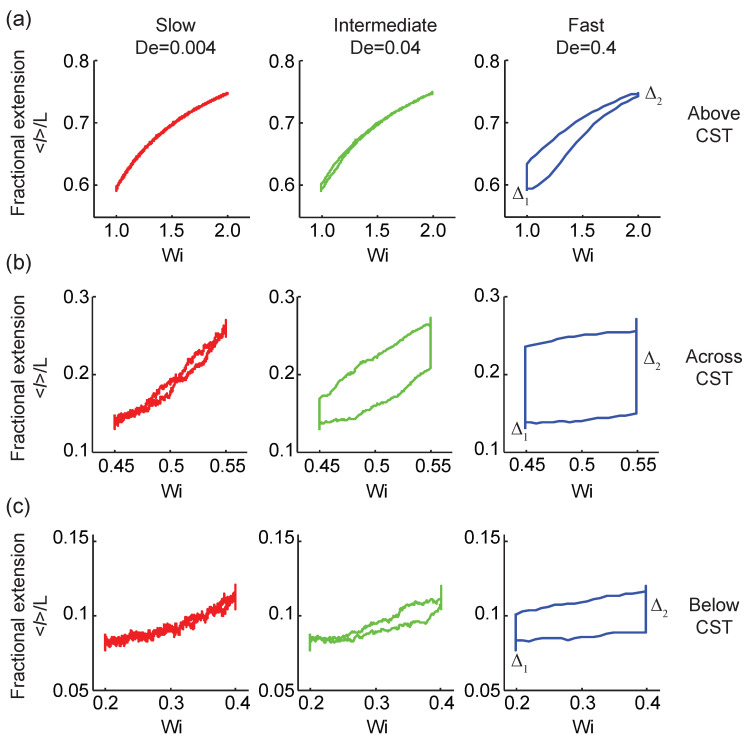
Rate-dependent viscoelastic polymer stretch loops in three distinct regimes with respect to the coil-stretch transition (CST). All trajectories correspond to the ensemble-averaged fractional extension of λ-DNA molecules perturbed with low, intermediate, and high transition rates (**a**) above the CST, from $Wi_{A}=1$ to $Wi_{B}=2$ (**b**) across the CST, from $Wi_{A}=0.45$ to $Wi_{B}=0.55$, and (**c**) below the CST, from $Wi_{A}=0.2$ to $Wi_{B}=0.4$.

We further studied transient polymer stretching dynamics using the Pipkin space framework [46], defined as a two-dimensional parameter space governed by the flow strength Wi and the transition rate De. As shown in Figure 3, no viscoelastic hysteresis is observed for polymers under extremely low rate transitions (De=0.004), and the viscoelastic stretch-strain rate loops collapse onto a single line. The polymer ensemble-averaged fractional extension is independent of the forward/backward transition path directions, regardless of flow strength. This suggests that at extremely slow transitions, the flow system responds in a quasi-equilibrium steady-state [39]. Under intermediate and fast transitions, however, the flow system responds in a nonequilibrium steady-state. We observe signatures of rate-dependent hysteresis in the ensemble-averaged polymer fractional extension. Importantly, these viscoelastic hysteresis loops suggest the existence of dissipation in the context of nonequilibrium steady-state thermodynamics [14].

We further probed viscoelastic hysteresis by studying polymer dynamics with initial and final Wi values across the CST (Figure 3b) and below the CST (Figure 3c). Figure 3b shows the ensemble-averaged polymer fractional extension in response to the time-dependent extensional flow protocol across the CST from $Wi_{A}=0.45$ to $Wi_{B}=0.55$. Our results show a strong rate-dependent hysteresis of the ensemble-average polymer conformation which increases as the transition rate is increased. Importantly, the hysteresis loops are more pronounced across the CST than above or below the CST, as shown in Figure 3a,c. This finding is consistent with the observation that molecular fluctuations, and hence thermodynamic dissipation, substantially increase near the CST [14]. At flow strengths below the CST ($Wi_{A}=0.2$ to $Wi_{B}=0.4$), viscoelastic hysteresis in the ensemble-averaged polymer fractional extension is again observed. The hysteresis loops become larger as the transition rate increases from De=0.004 to De=0.4 (Figure 3c).

The rate-dependent viscoelastic stretch-strain loops reveal information regarding the fundamental nature of the CST. At flow strengths above the CST, the coil-lag $\Delta_{1}$ at the lower flow strength ($Wi_{A}=1$) is larger than the stretch-lag $\Delta_{2}$ at a higher flow strength ($Wi_{B}=2$), as shown in Figure 2 and Figure 3a. This behavior can be rationalized in terms of the effective conformational energy of single polymer chains [27,36]. Above the CST, the effective conformational energy of the polymer chain has a single-valued minimum corresponding to the stretched polymer state, hence the polymer chain prefers to equilibrate to a more extended steady-state after flow deformation, leading to $\Delta_{2}\leq \Delta_{1}$. Whereas under quasi-equilibrium steady-state conditions, the viscoelastic hysteresis loops collapse onto a single curve, $\Delta_{1}=\Delta_{2}=0$. Below the CST, we observe a different relationship between the coil- and stretch-lag when compared with our observations above the CST. Below the CST, $\Delta_{2}\geq \Delta_{1}$ (Figure 3c), suggesting that polymers prefer to equilibrate to a more coiled steady-state after flow deformation because the effective polymer conformational energy has a single minimum corresponding to the coiled polymer state. In the vicinity of the CST, the effective polymer conformational energy is flattened out, suggesting an increase in polymer chain fluctuations near a critical point [27,34,36]. Hence, across the CST, the polymer chain is able to equilibrate to both the coiled and stretched states after flow deformation, depending on its initial state. Overall, the flow-dependent behavior or $\Delta_{1}$ and $\Delta_{2}$ generally depends on the initial flow strength $Wi_{A}$, the final flow strength $Wi_{B}$, and the transition rates De.

### 3.2. Determination of Free Energy from Work Distributions

In the context of fluctuation theorems, the time-dependent extensional flow protocol used in this work allows for the determination of nonequilibrium thermodynamic quantities from forward and backward work distributions. Here, we utilize a work definition that allows for the determination of an effective free energy, $F^{*}$, from fluctuation theorems including the Crooks relation [9,14]. This definition provides the amount of work performed on the system due to the changes in the control parameter, Wi. Note that this work definition does not account for ‘housekeeping work’ [14], which is the work required to continuously maintain a polymer chain in flow at a constant flow strength at the desired nonequilibrium steady state. As a result, the work values may be negative depending on the amount of energy required to maintain the steady state.

The corresponding definition of work for a polymer chain (dumbbell model) in planar extensional flow is [14]:

(3) $$\begin{array}{c} W=-\int_{t_{A}}^{t_{B}}\frac{1}{\tau_{R}}\frac{dWi}{dt^{\prime }}\left(x_{1}^{2}-x_{2}^{2}\right)dt^{\prime } \end{array}$$

where $x=r_{2}-r_{1}$ represents the dimensionless end-to-end connector vector, and the subscripts 1 and 2 for x represent chain orientation along the extensional and compressional axes, respectively. The end-to-end distance is non-dimensionalized using a characteristic length scale $l_{s}=\sqrt{k_{B}T/H_{s}}$, where $k_{B}T$ is the thermal energy and $H_{s}$ is the Hookean spring force constant (the Hookean spring force constant is $H_{s}=3k_{B}T/N_{k,s}{b_{k}}^{2}$. The Kuhn step size is $b_{k}$, and the number of Kuhn steps per entropic spring is $N_{k,s}$. For fluorescently labeled DNA molecules, $b_{k}=0.132$ μm and $N_{k,s}=159$ for the dumbbell model for λ−DNA). Physically, Equation (3) allows for determination of the work required to transition a polymer chain from $Wi_{A}$ to $Wi_{B}$. The quantities *t* and the transition rate dWi/dt in Equation (3) are non-dimensionalized using the time constant for a Hookean dumbbell, $\lambda_{H}=\zeta /4H_{s}$, and ζ is the hydrodynamic drag coefficient on each bead of the dumbbell (the bead drag coefficient is $\zeta =12k_{B}T\tau_{R}/0.9N_{k,s}{b_{k}}^{2}$, and the value of ζ is determined based on the longest polymer relaxation time $\tau_{R}$ from single molecule experiments, as discussed in prior work [14]).

**Figure 4:**
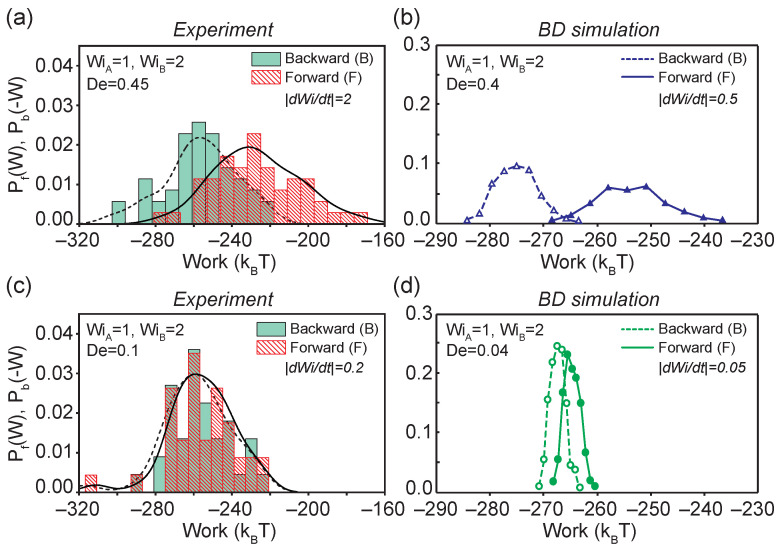
Probability density functions of forward and backward work distributions for transitions that occur *above* the coil-stretch transition ($Wi_{A}=1.0$ to $Wi_{B}=2.0$) at a fast transition rate of De∼0.4 from (**a**) single molecule experiments and (**b**) BD simulations, and at slow transition rate of De∼0.1 from (**c**) single molecule experiments and (**d**) BD simulations. The experimental ensemble contain n=50, and n=38 molecular traces for De=0.45 and De=0.1, respectively. All simulation ensembles contain n=500 molecular traces.

Using Equation (3), the work required to perform forward (backward) flow transitions on single polymer molecules corresponding to moving along points b(d) to c(e) is determined, as shown in Figure 1a. In all cases, the system is allowed to reach the non-equilibrium steady-state before inducing changes in the flow strength (a→b and c→d in Figure 1a), which satisfies a crucial requirement for applying the CFT. Using this approach, we first determined the forward $P_{F}\left(W\right)$ and backward $P_{B}\left(-W\right)$ work distributions for DNA molecules transitioning from nonequilibrium steady-states with flow strengths $Wi_{A}=1$ to $Wi_{B}=2$ at De≈0.45 and De≈0.1 from single molecule experiments and BD simulations (Figure 4).

The work distributions show a strong dependence on transition rates between the initial and final flow strengths, as shown in Figure 4a,b. Broad work distributions for both forward and backward processes were observed at a fast transition rate of De≈0.4 (Figure 4a,b). By applying the CFT (Equation (1)), which corresponds to choosing the work value where the $P_{F}\left(W\right)$ and $P_{B}\left(-W\right)$ intersect, we determine the effective free energy difference, $\Delta F^{*}$, between the two nonequilibrium steady-states from measurable quantities in flow. For the process shown in Figure 4a,b, $\Delta F^{*}$ was determined from single molecule experiments as $\Delta F^{*}=-242.5\;k_{B}T$ and from BD simulations as $\Delta F^{*}=-266.4\;k_{B}T$. The average forward work 〈W〉 is approximately 30 $k_{B}T$ larger than the average backward work 〈−W〉 from experiments (or 20 $k_{B}T$ from BD simulations). These results indicate the nature of the strongly irreversible process and energy losses between the two stretched states of single polymers above CST at De≈0.4, which is consistent with the observations in viscoelastic hysteresis loops (Figure 3a). In contrast to the broad work distributions at De≈0.4, the work distributions at an intermediate transition time of De≈0.1 drastically narrow down with small difference in 〈W〉 and 〈−W〉 (Figure 4c,d). At De≈0.1, CFT predicts $\Delta F^{*}=-259.8\;k_{B}T$ from single molecule experiments and $\Delta F^{*}=-265.9\;k_{B}T$ from BD simulations. In general, our results show qualitative agreement between single molecule experiments and BD simulations in predicting the forward and backward work distributions, and near quantitative agreement in determining the effective free energy.

**Figure 5:**
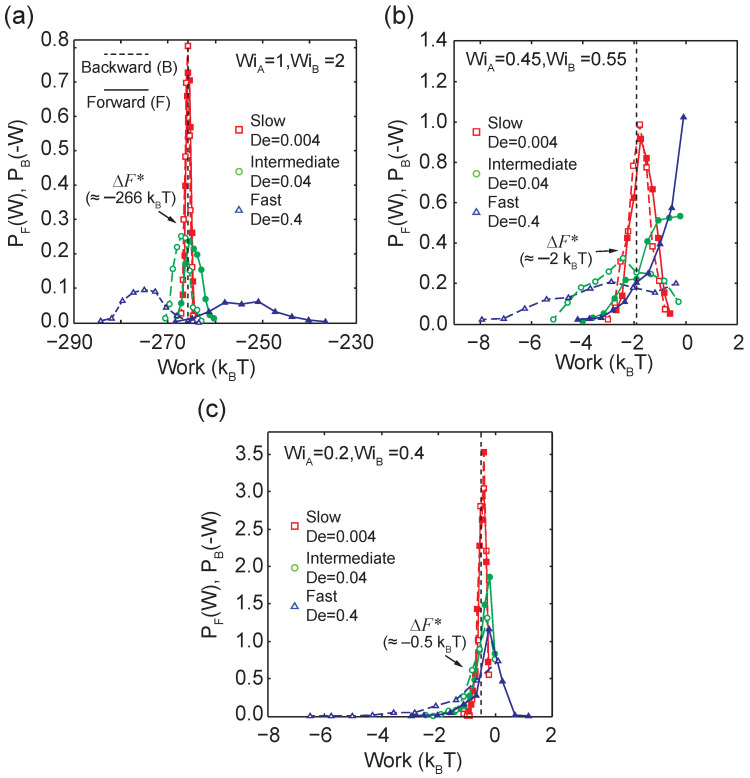
Probability distribution functions of forward and backward work distributions from BD simulations for transitions that occur: (**a**) *Above* the coil-stretch transition ($Wi_{A}=1.0$ to $Wi_{B}=2.0$), (**b**) *across* the coil-stretch transition ($Wi_{A}=0.45$ to $Wi_{B}=0.55$), and (**c**) *below* the coil-stretch transition ($Wi_{A}=0.2$ to $Wi_{B}=0.4$).

We further investigated work distributions at an extremely slow transition rate of De≈0.004 using BD simulations, as shown in Figure 5a. Here, the system resides in quasi-equilibrium steady-states given the slow transition rates. The work distributions are narrow compared to faster transition rates De≈0.4 and De≈0.04, and the average work performed in the forward and backward directions is approximately equal, such that $\langle W\rangle \approx \langle -W\rangle \approx -266\;k_{B}T$. The differences in 〈W〉 and 〈−W〉 are within $\pm 2\;k_{B}T$ and $\Delta F^{*}=-265.8k_{B}T$. Remarkably, we recover nearly the same effective free energy difference between the two states ($Wi_{A}=1$ and $Wi_{B}=2$) at three different transition rates (De≈ 0.4, 0.04, and 0.004) spanning two orders of magnitude, with effective free energy values of −266.4 $k_{B}T$, −265.9 $k_{B}T$, and −265.8 $k_{B}T$, respectively. In principle, the CFT is valid regardless of the irreversibility during transitions.

To further demonstrate the generality of CFT for nonequilibrium steady-states in single polymer dynamics, we consider transitions across and below the CST at different transition rates (Figure 5b,c). Our results show a broadening in the forward and backward work distributions when the transition time decreases from De≈0.004 to De≈0.4, which is consistent with results from transitions above the CST. Interestingly, the effective free energy differences between nonequilibrium steady states drastically decrease from $\Delta F^{*}\approx -266\;k_{B}T$ above CST ($Wi_{A}=1$ and $Wi_{B}=2$) to $\Delta F^{*}\approx -0.5\;k_{B}T$ below the CST. This can be rationalized because in a highly stretched state, a polymer chain significantly loses conformational entropy, and more work is required to transition the polymers into a highly stretched state compared to simply maintaining a coiled state conformation.

Finally, we note that the analysis presented here provides a *nonequilibrium* demonstration of the CFT, which allows for the determination of the effective free energy $F^{*}$ but not the nonequilibrium Helmholtz-free energy *F*. This is in contrast with the *equilibrium* demonstration of CFT, which allows for determination of the equilibrium Helmholtz-free energy [17]. However, the CFT can be easily recast in a form that allows for the direct determination of *F* by noting that $F=F^{*}-\langle \chi \rangle$ [14], where −〈χ〉 is a flow energy that maintains the system out-of-equilibrium. For a polymer dumbbell in planar extensional flow, the dimensionless flow energy $\chi =-Wi\left(x_{1}^{2}-x_{2}^{2}\right)/\tau_{R}$ [14].

### 3.3. Validity of the Crooks Fluctuation Theorem

**Figure 6:**
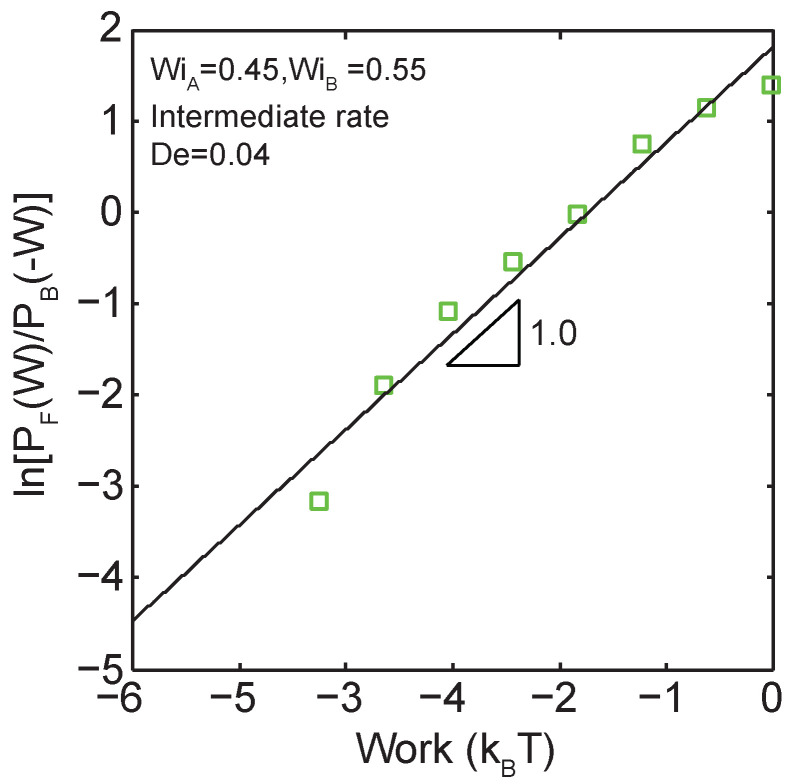
Demonstration of a linear relationship between the log ratio of forward and backward work distributions as a function of work for transitions across the coil-stretch transition ($Wi_{A}=0.45$ to $Wi_{B}=0.55$) at an intermediate transition time of De≈0.04.

We further validate the application of the Crooks fluctuation theorem to single polymer dynamics. As predicated by the CFT (Equation (1)), the log ratio of forward and backward probabilities of work are predicted to follow a linear relationship as a function of the total work done during the transition. Indeed, as indicated in Figure 6, a linear relation is observed for the log ratio of forward and backward work probabilities versus the work performed during the transition between two nonequilibrium steady-states ($Wi_{A}=0.45$ to $Wi_{B}=0.55$) at De≈0.04.

## 4. Conclusions

In this article, we use a combination of single molecule experiments and Brownian dynamics (BD) simulations to explore the existence of a generalized polymer viscoelastic hysteresis at the molecular level. The Crooks fluctuation theorem (CFT) is applied to analyze nonequilibrium steady-states (NESSs) of polymer chains based on a time-dependent planar extensional flow protocol. Reasonable agreement is observed between experiments and simulations in the context of free energy values describing the forward and reverse flow processes. Our results showed that viscoelastic hysteresis arises from the different transition rates De between NESSs in our flow protocol and exists over a wide range of imposed flow strengths Wi. Using BD simulations, we found that such viscoelastic hysteresis exists even in the absence of the intramolecular hydrodynamic interactions. Furthermore, we introduced two new quantities, the ‘coil-lag’ $\Delta_{1}$ and the ‘stretch-lag’ $\Delta_{2}$, which characterize the viscoelastic hysteresis loop for single polymers above, across, and below the coil-stretch transition.

Based on the time-dependent planar extensional flow protocol, we determine the work distributions of single polymers transitioning between two nonequilibrium steady-states with different flow strengths $Wi_{A}$ and $Wi_{B}$ under varying transition rates De. In this way, we are able to perform nonequilibrium free energy recovery using CFT. Remarkably, our results show that the free energy differences can be determined with reasonable accuracy regardless of transition rates spanning three orders of magnitude. In contrast to prior work using CFT to determine free energies from a non-thermodynamic flowing system [19], our work demonstrates the determination of free energy using the CFT from a true flowing system consisting of dilute polymer chains in a time-dependent flow. Based on our demonstrated flow protocol and analysis method, the free energy difference of polymers under various flow strengths can be determined from work distributions. Moving forward, beyond linear polymer chains in dilute solutions, this framework can be applied to investigate the flow dynamics of topologically complex polymers in nondilute solutions [45,47,48].

## Data Availability

The data presented in this study are available on request from the corresponding author. The data are not publicly available due to privacy.
